# Lateral extra‐articular tenodesis preserves quadriceps strength without significantly affecting plyometric performance after anterior cruciate ligament reconstruction

**DOI:** 10.1002/jeo2.70456

**Published:** 2025-10-09

**Authors:** Mihail Lazar Mioc, Sarah Crosbie, Blaithin Brady, Mihai Vioreanu

**Affiliations:** ^1^ Department XV Orthopedics and Traumatology Victor Babes University of Medicine and Pharmacy Timisoara Romania; ^2^ UPMC Sport Surgery Clinic Dublin Ireland

**Keywords:** ACL reconstruction, ACL rehabilitation, lateral extra‐articular tenodesis, quadriceps muscle strength, reactive strength index, return to sport

## Abstract

**Purpose:**

To investigate whether adding lateral extra‐articular tenodesis to anterior cruciate ligament reconstruction affects quadriceps strength or reactive strength at 6–8 months postoperatively in young, high‐risk athletes.

**Methods:**

This retrospective cohort study analysed patients who underwent primary anterior cruciate ligament reconstruction with or without lateral extra‐articular tenodesis between 2015 and 2019. All surgeries were performed using hamstring tendon autografts by a single surgeon. Patients completed standardised isokinetic and 3D biomechanical testing between 6 and 8 months postoperatively. Quadriceps strength was assessed using isokinetic dynamometry, with results expressed as peak torque relative to body weight and limb symmetry index. Reactive strength was measured through double‐leg and single‐leg drop jump tests in a motion analysis laboratory. The reactive strength index was calculated by dividing jump height by ground contact time. Group comparisons were performed using independent t‐tests or Mann–Whitney *U* tests, with significance set at *p* < 0.05.

**Results:**

Sixty‐eight patients were included: 39 underwent anterior cruciate ligament reconstruction with lateral extra‐articular tenodesis and 29 underwent isolated anterior cruciate ligament reconstruction. Quadriceps strength was similar between groups for both limbs and for limb symmetry index. Reactive strength values were lower in the lateral extra‐articular tenodesis group, but the differences were not statistically significant. A non‐significant trend toward lower reactive strength was observed in lateral extra‐articular tenodesis patients under 18 years of age.

**Conclusions:**

Lateral extra‐articular tenodesis does not impair quadriceps strength recovery at 6–8 months following anterior cruciate ligament reconstruction. While reactive strength trended lower in these patients, the difference was not statistically significant. Further investigation is warranted to clarify its clinical relevance.

**Level of Evidence:**

Level III.

**Abbreviations**
ACLR: anterior cruciate ligament reconstruction
ACLR‐LET: anterior cruciate ligament reconstruction with lateral extra‐articular tenodesis
ALLR: anterolateral ligament reconstruction
CACL: contralateral anterior cruciate ligament
CI: confidence interval
CMJ: counter‐movement jump
DJ: drop jump
DLDJ: double leg drop jump
HT: hamstring tendons
LET: lateral extra‐articular tenodesis
LSI: limb symmetry index
RSI: reactive strength index
RTS: return to sport
SLDJ: single leg drop jump

## INTRODUCTION

Anterior cruciate ligament (ACL) injuries account for approximately 50% of all sports‐related knee injuries, with an annual incidence of 3% in amateur athletes and 15% in elite athletes [35, 39]. Given the high rate of ACL injuries, surgical reconstruction remains the gold standard for young, active individuals engaged in pivoting sports [15]. The most commonly used autografts for ACL reconstruction (ACLR) include hamstring tendons (HT) and bone‐patellar tendon‐bone (BTB) [31].

Despite continuous advancements in ACLR techniques, isolated intra‐articular reconstruction often fails to restore knee rotational stability fully, leaving athletes vulnerable to re‐injury [1, 8]. Reports indicate ACL graft rupture rates from 6% to 25%, while contralateral ACL (CACL) injuries occur in 7% to 20% of cases within 2–7 years postoperatively [14, 20]. Several factors contribute to an increased risk of ACL re‐rupture, including high‐level participation in cutting and pivoting sports, ligamentous hyperlaxity, younger age (<25 years old), increased tibial slope, revision surgery and a pronounced pivot shift test [4, 9, 27, 32]. Emerging evidence also suggests that biological factors such as vitamin D status may influence both ACL injury susceptibility and postoperative recovery [29].

To mitigate residual rotational instability and reduce graft failure rates, interest in lateral extra‐articular tenodesis (LET) as an adjunct to ACLR has resurged. Systematic reviews and meta‐analyses indicate that ACLR‐LET effectively controls rotatory stability and lowers re‐rupture rates [2, 3, 4, 6, 10, 13, 15, 24, 33, 37]. However, concerns remain regarding its potential impact on quadriceps function, as the additional surgical injury may lead to greater quadriceps strength deficits [9].

Strength deficits following ACLR have significant implications for return to sport (RTS) decisions. The limb symmetry index (LSI), a measure of inter‐limb strength balance, is widely used to assess recovery, with values below 90% associated with increased re‐injury risk. Kyritsis et al. showed that athletes who fail to meet RTS criteria (quadriceps strength deficit <20% and hop test limb symmetry index >90%) had a fourfold increased risk of ACL graft rupture [20]. While quadriceps strength and single‐leg hop tests are widely used RTS benchmarks, recent evidence suggests that reactive strength may be a more sensitive predictor of neuromuscular recovery [26]. Reactive strength refers to an athlete's ability to rapidly transition from eccentric to concentric muscle contraction during stretch‐shortening cycle movements, such as drop jumps. It is typically quantified using the reactive strength index (RSI), calculated as jump height divided by ground contact time. Unlike traditional hop tests, which reflect maximal effort or distance, RSI captures the neuromuscular efficiency of explosive movements and may provide better insight into sport‐specific readiness [12].

A systematic review by Ashgibi et al. highlighted that isokinetic knee extension strength and single‐leg hop tests have the highest predictive value for ACL re‐injury risk [1]. However, there is limited research on plyometric performance and reactive strength outcomes in ACLR‐LET patients. Understanding whether ACLR‐LET compromises neuromuscular readiness for RTS could help refine rehabilitation protocols and surgical decision‐making.

This study aimed to investigate differences in maximal quadriceps strength and plyometric activity between isolated ACLR and ACLR‐LET athletes, using isokinetic dynamometry and 3D biomechanical testing at 6–8 months postoperatively. We hypothesised that there would be no reduction in quadriceps strength or reactive strength in ACLR‐LET patients compared to isolated ACLR.

## MATERIAL AND METHODS

### Study design

**Figure 1:**
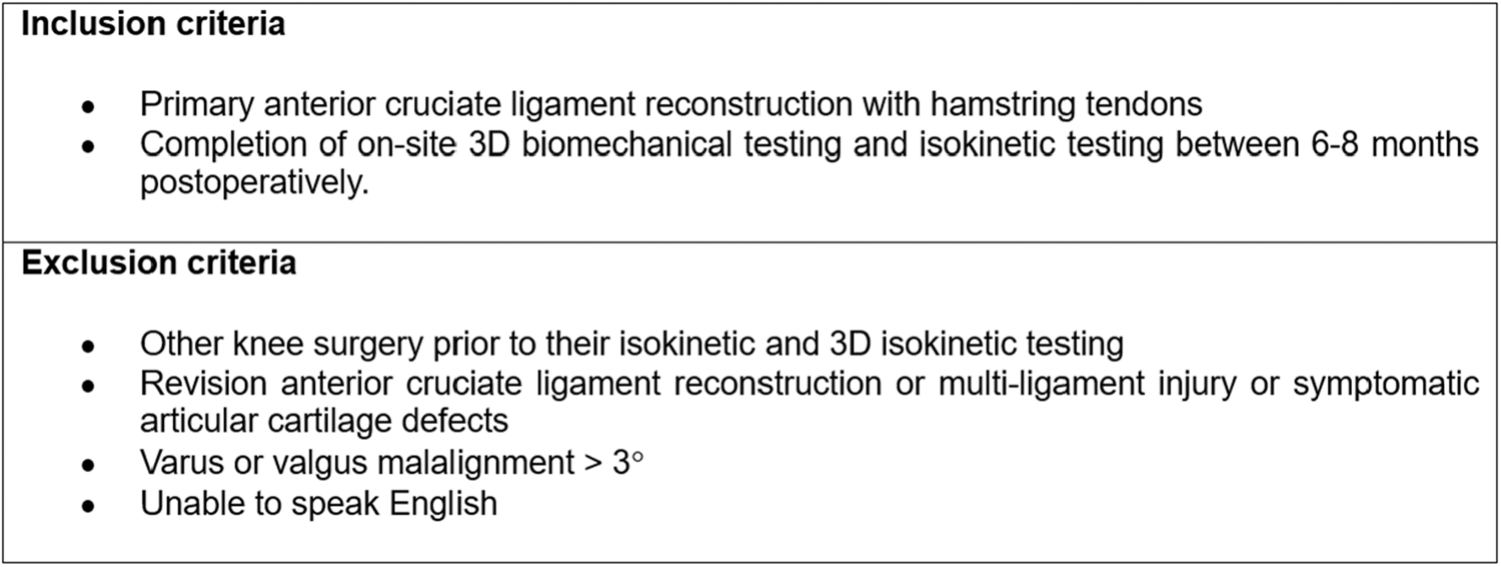
Study inclusion and exclusion criteria.

**Figure 2:**
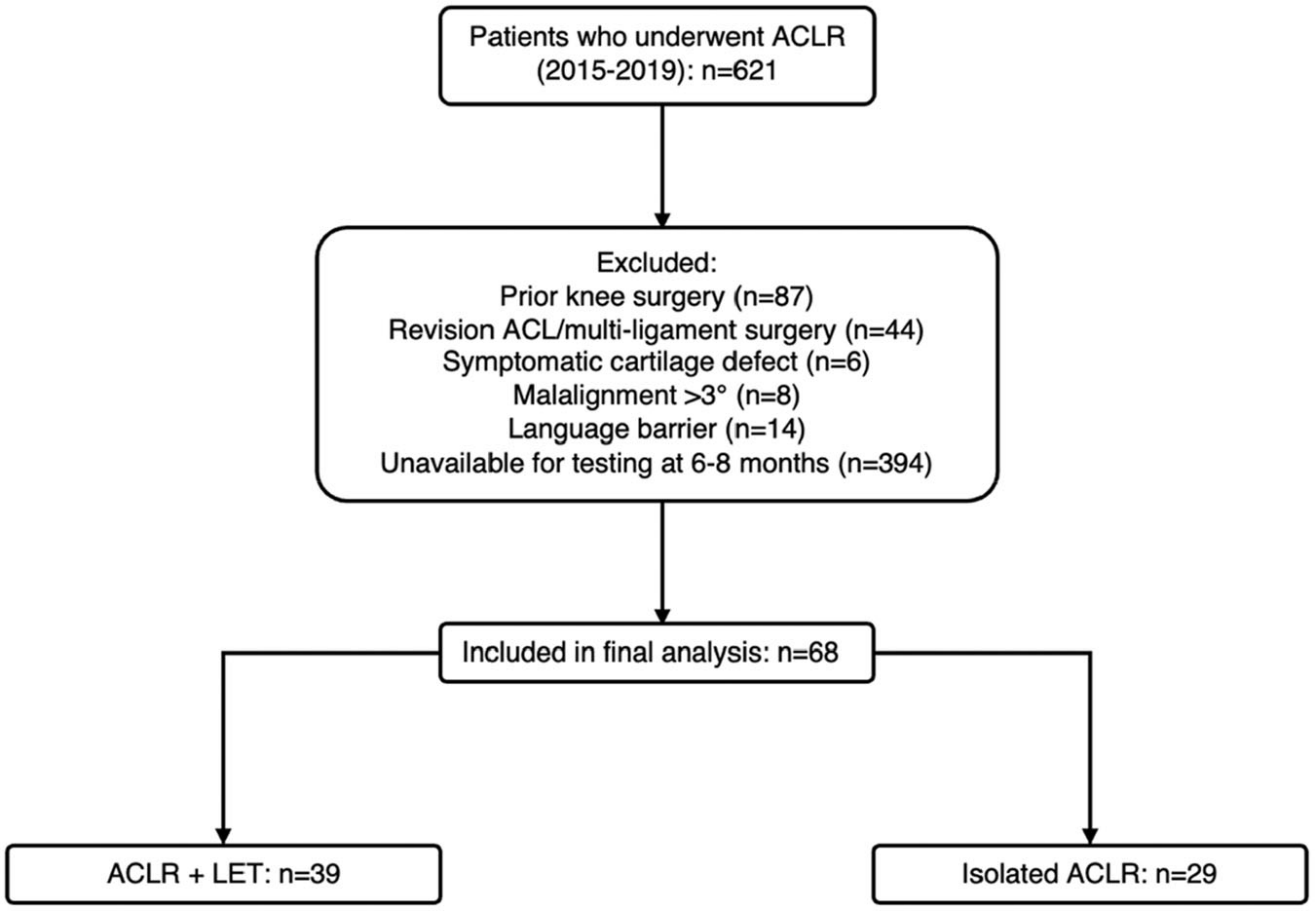
Study cohort selection and exclusion pathway for patients undergoing ACLR between 2015 and 2019. ACLR, anterior cruciate ligament reconstruction; LET, lateral extra‐articular tenodesis.

This retrospective cohort study analysed prospectively gathered data from a single‐surgeon series of ACLRs performed between 2015 and 2019. Eligible participants were identified retrospectively from the institution's database based on specific inclusion and exclusion criteria, as shown in Figure 1. This study was conceived adhering to the STROBE (Strengthening the Reporting of Observation Studies in Epidemiology) guidelines to ensure transparent and accurate reporting of observational research [36]. The STROBE checklist was used to structure the study design, data collection, analysis, and reporting. This study was performed in line with the Declaration of Helsinki. Explicit consent to participate was not required as retrospective chart reviews are considered low‐risk studies under institutional guidelines, and approval from our institution's ethics committee was obtained per reference number MiVi_SSC_2021_ACLR. No funding was received during the preparation of this manuscript. A detailed flow diagram of patient selection and group allocation is presented in Figure 2.

**Figure 3:**
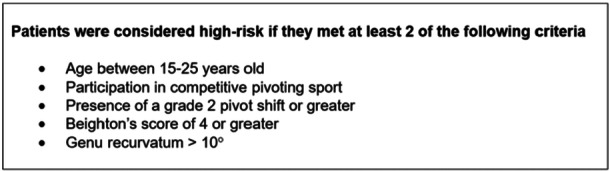
Selection criteria for anterior cruciate ligament reconstruction with lateral extra‐articular tenodesis (ACLR ‐ LET).

The ACLR‐LET was indicated for patients who fell into the high‐risk category, at the discretion of the surgeon (Figure 3). All patients underwent primary ACLR using an HT graft from the ipsilateral limb. For those receiving the addition LET, the modified Lemaire technique was used [40].

### Testing procedures

Testing was conducted at 6–8 months postoperatively, as this period represents a critical phase of RTS decision‐making. At this stage, athletes are typically cleared for sport‐specific training, but full RTS often occurs 10–12 months postoperatively to reduce the risk of re‐injury. Prior research suggests that RTS readiness criteria, including quadriceps strength and reactive strength, are best assessed in this transitional phase. Both reactive strength testing and isokinetic strength testing assessments have demonstrated high intra‐rater reliability in previously published studies [7, 11].

Testing was conducted at a specialised sports medicine laboratory using an 8‐camera 3D motion analysis system (Bonita B10, Vicon) synchronised with dual force platforms (BP400600, AMTI) to measure ground contact time. Vicon Nexus software (version 1.8.2) captured and processed force data at 1000 Hz. Participants performed drop jumps from a standardised height, wearing athletic footwear and keeping hands on hips throughout to control for arm swing. Each athlete completed three practice trials, followed by three maximal‐effort recorded trials per leg, beginning with the non‐injured limb. Jump height was calculated using the impulse–momentum method, and the RSI was computed as jump height divided by ground contact time [12, 21]. For clarity, RSI from the double‐leg drop jump is referred to as RSI, while single‐leg values are reported as RSI‐A (right limb) and RSI‐B (left limb), consistent with our data recording convention.

Following the reactive strength tests, isokinetic dynamometry testing was carried out. This is a validated and reliable quadriceps strength testing [18, 30, 34]. Each participant performed a standardised 5‐min warm‐up, and then the non‐ACL reconstructed limb was tested first. Isometric dynamometry assessment was conducted with the aid of the Humac Norm Isokinetic Extremity System. A protocol of five repetitions of concentric knee extension at an angular velocity of 60°/s from 0° to 100° of knee flexion using gravity correction was used. Participants had a warm‐up set of five repetitions to familiarise themselves with the test procedure and the protocol before completing the test. Peak torque relative to bodyweight ((peak torque/bodyweight) × 100) was the variable of interest for knee extension. The limb symmetry index was then calculated for quadriceps strength by dividing the ACLR limb score by that of the non‐ACLR limb, multiplying the outcome by 100, according to guidelines published in a previous systematic review [35].

### Statistical analysis

The Shapiro–Wilk test was used to assess the normality of distribution for the dependent variables. Due to a mix of normal and non‐normal distributions, continuous dependent variables were analysed using independent t‐tests or Mann–Whitney *U*‐tests. No a priori sample size calculation was performed, as this study retrospectively included all eligible patients who met the inclusion criteria between 2015 and 2019. The availability of complete biomechanical and isokinetic testing data within the specified follow‐up period determined the sample size. Depending on the statistical test applied, data were reported as mean and standard deviation for parametric variables or median and interquartile range for non‐parametric variables. No missing data were present for the primary outcome variables. All included participants completed full biomechanical and isokinetic testing. Sensitivity analyses were not performed, as the primary comparisons were predefined and based on complete data. A *p*‐value of < 0.05 was considered statistically significant. All statistical analyses were performed using IBM SPSS Statistics version 27.

## RESULTS

A total of 68 patients were included in the study, with 39 (57%) undergoing ACLR‐LET and 29 (43%) undergoing isolated ACLR. The mean age of participants was 20.5 years (±5.2), ranging from 12 to 36 years. The distribution of gender, meniscal injury, and limb dominance was comparable between groups. Further descriptive cohort data can be found in Tables 1 and 2. No missing data were present for any of the primary outcome variables, and complete isokinetic biomechanical testing data were available for all 68 included participants.

**Table 1:** Baseline cohort characteristics.

Measure	ACLR	ACLR‐LET
No. of patients	29	39
Mean age (SD; range)	22.21 (5.34; 14–36)	18.9 (4.76; 12–32)
Male/female (%)	23/6 (79%/21%)	24/15 (62%/38%)
Meniscal involvement (%)	24 (83%)	31 (79%)

Abbreviations: ACLR‐LET, anterior cruciate ligament reconstruction with lateral extra‐articular tenodesis; SD, standard deviation.

**Table 2:** Sample cohort demographics.

Measures (*N* = 68)	Mean (SD)
Peak torque relative to BW* Quad injured	217.66 (45)
Peak torque relative to BW* Quad non‐injured	243.76 (39.8)
Quad LSI**	89.03 (9.6)
RSI***	0.82 (0.3)
RSI ‐ Injured limb***	0.26 (0.11)
RSI ‐ Uninjured limb***	0.33 (0.12)

Abbreviations: ACLR, anterior cruciate ligament reconstruction; LSI, limb symmetry index; RSI, reactive strength index; SD, standard deviation.

* Measured in (Nm/body weight) × 100;

** Measured as (ACLR limb score/non‐ACLR limb score) × 100;

*** Measured as jump height/ground contact time.

**Figure 4:**
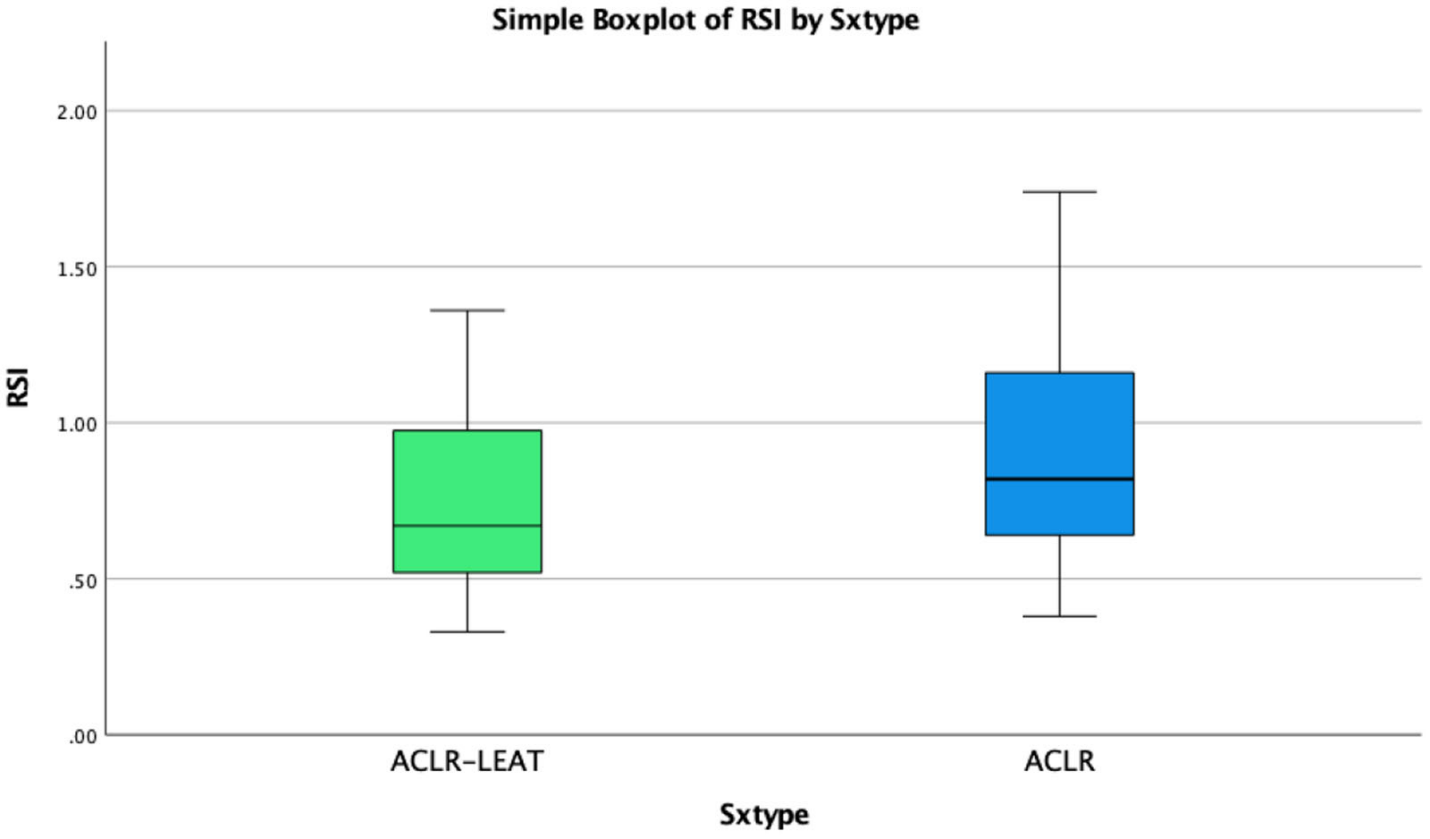
Boxplot of RSI in ACLR‐LET and ACLR groups by type of surgery (Sxtype). The ACLR‐LET group shows a lower median RSI compared to isolated ACLR, with a narrower interquartile range. Whiskers indicate data variability within each group. ACLR‐LET, anterior cruciate ligament reconstruction with lateral extra‐articular tenodesis; RSI, reactive strength index.

RSI values were lower in the ACLR‐LET group; however, these differences were not statistically significant at the time of RTS testing (Table 3). The visual representation of the median RSI scores between groups is displayed in Figure 4.

**Table 3:** Reactive strength index (RSI) outcomes.

Measure	ACLR‐LET	ACLR	*p* value	Effect size	95% CI
RSI (Double‐leg drop jump)	0.67 (IQR: 0.47–0.83)	0.82 (IQR: 0.58–1.02)	0.41	*r* = 0.1	N/A
RSI ‐ Injured limb	0.21 (IQR: 0.15–0.34)	0.24 (IQR: 0.18–0.35)	0.43	*r* = 0.1	N/A
RSI ‐ Uninjured limb	0.37 (IQR: 0.21–0.43)	0.32 (IQR: 0.26–0.39)	0.36	*d* = 0.23	−0.03 to −0.09

*Note*: RSI values are presented as median (interquartile range). Comparisons were performed using the Mann–Whitney *U* test for non‐parametric data (RSI and RSI – injured limb) and the independent t‐test for parametric data (RSI – uninjured limb). Effect sizes are reported as *r* for Mann–Whitney *U* tests (calculated as *Z*/√*N*) and Cohen's *d* for t‐tests. Statistical significance was set at *p* < 0.05.

Abbreviations: ACLR‐LET, anterior cruciate ligament reconstruction with lateral extra‐articular tenodesis; CI, confidence interval; IQR, interquartile range.

There was no statistically significant difference in peak quadriceps torque between groups (Table 4). These findings indicate that quadriceps strength recovery was similar between groups at 6–8 months postoperatively.

**Table 4:** Quadriceps strength outcomes.

Measure	ACLR‐LET	ACLR	*p* value	95% CI
Peak torque (injured limb)	216.5 ± 45.7 Nm/kg	219.2 ± 44.9 Nm/kg	0.81	−15.7 to 19.3
Peak torque (uninjured limb)	250 ± 62 Nm/kg	250 ± 59 Nm/kg	0.68	−22.5 to 18.7
Limb Symmetry Index (LSI)	88.95 ± 7.96%	89.14 ± 12.09%	0.94	−5.2 to 5.6

*Note*: LSI was calculated as the ACLR limb score divided by the non‐ACLR limb score × 100. Group comparisons were performed using independent t‐tests, with *p*‐values and 95% confidence intervals (CIs) reported. Statistical significance was set at *p* < 0.05.

Abbreviation: ACLR‐LET, anterior cruciate ligament reconstruction with lateral extra‐articular tenodesis.

Subgroup analysis revealed no significant interactions between sex (male vs. female) or meniscal involvement (with vs. without meniscal injury) for quadriceps strength or RSI outcomes (*p* > 0.05 for all comparisons). However, a trend toward lower RSI values in ACLR‐LET athletes under 18 years old was observed (*p* = 0.07), suggesting that younger athletes may experience greater neuromuscular deficits following ACLR‐LET (Figure 4). Although this trend did not reach statistical significance, it may warrant further investigation in future studies.

## DISCUSSION

This study investigated differences in maximal quadriceps strength and reactive strength (plyometric ability) between patients undergoing isolated ACLR versus ACLR‐LET at 6–8 months postoperatively. As hypothesised, there were no significant differences in quadriceps strength between groups, suggesting that ACLR‐LET does not impair maximal strength recovery at this stage of rehabilitation. However, contrary to our hypothesis, reactive strength was significantly lower in ACLR‐LET patients across all RSI outcomes, indicating a possible delay in plyometric function recovery before return to sport (RTS).

### Isokinetic strength

Our findings align with previous research demonstrating that ACLR‐LET does not negatively impact quadriceps strength. Oni et al. examined isokinetic strength following ACLR, but their comparison focused on isolated intra‐articular versus extra‐articular grafts only, limiting its relevance to our study population [28]. Marcacci et al. prospectively analysed 50 athletes following ACLR‐LET using a modified Lemaire procedure and found similar knee extension strength between injured and uninjured limbs, with LSI values below 10% for knee flexor strength [25]. However, their study lacked a control group and included a heterogeneous sex distribution, making direct comparisons difficult.

More recently, Joseph et al. retrospectively compared isokinetic strength in ACLR‐LET versus ACLR alone in 35 and 51 patients, respectively. They found no significant differences in quadriceps strength when tested at 240°/s and 90°/s at 6 months postoperatively [16]. Our results reinforce their findings, particularly as our study cohort consisted of a younger, more athletic population with a more balanced sex distribution and meniscal involvement between groups. Similarly, another study reported similar isokinetic strength differences post‐ACLR, resembling our observed ACLR‐LET deficits when tested at 60°/s [41].

In contrast, the STABILITY trial by Getgood et al. reported that ACLR‐LET patients exhibited significantly lower quadriceps peak torque and average power at 6 months compared to isolated ACLR. However, these differences became non‐significant by 12 months and were absent by 24 months [8]. Since our study assessed strength at 6–8 months postoperatively, it remains unclear when ACLR‐LET patients achieve strength parity with isolated ACLR. Given that RTS typically occurs between 9 and 12 months, our findings suggest that quadriceps strength is not a limiting factor for RTS in ACLR‐LET patients. Further reinforcing this, Weaver et al. recently demonstrated that adolescent patients undergoing ACLR‐LET exhibited significantly lower quadriceps strength at 6–9 months postoperatively compared to isolated ACLR. Their study found a 25% reduction in involved limb knee extension strength in ACLR‐LET patients, supporting prior work [38]. This suggests that lateral extra‐articular procedures may introduce additional neuromuscular deficits, potentially delaying RTS in younger athletes.

### Reactive strength

To our knowledge, this is the first study to compare RSI between ACLR‐LET and isolated ACLR patients prior to RTS. Reactive strength reflects an athlete's ability to generate force quickly following an eccentric load, which is critical for explosive sports movements [12, 19, 28]. While ACLR‐LET patients showed a trend toward lower RSI values in both double‐leg and injured limb drop jump tests, these differences were not statistically significant.

Existing literature on RSI post‐ACLR remains limited. Maestroni et al. performed a systematic review and meta‐analysis of RSI in adult males following intra‐articular ACLR, but only one study met their inclusion criteria [23]. King et al. assessed 156 multidirectional athletes at 9 months post‐ACLR and reported reduced RSI in the ACLR limb compared to the uninjured limb, though the study lacked a control group [17]. Another study compared ACLR vs. healthy controls in a cross‐sectional design and found no functional RSI deficits between groups when testing single‐leg hop, 6 m timed hop, squat jump, countermovement jump (CMJ), drop jump (DJ), and rebound jump. However, the small sample size (*n* = 10 ACLR, 8 males and 2 females) and testing timeline (≥ 2 years post‐surgery) limit direct comparisons to our study [7].

One possible explanation for reduced RSI in ACLR‐LET patients is altered neuromuscular control due to lateral soft tissue trauma. Delahunt et al. suggested that altered sensorimotor control and proprioception post‐ACLR influence lower limb kinematics during plyometric tasks. Given that ACLR‐LET involves an additional lateral incision and greater surgical morbidity, it is likely that neuromuscular disruptions are more pronounced in this cohort [5, 10]. Although our data did not reach statistical significance, the observed trends warrant further investigation. Since reactive strength is largely governed by subconscious neural reflexes [22, 23], longer recovery periods may be necessary to restore optimal plyometric function in ACLR‐LET athletes.

While Weaver et al. did not specifically analyse RSI, their findings highlight the potential for delayed neuromuscular recovery in ACLR‐LET patients. Given that quadriceps strength plays a key role in force absorption and propulsion during plyometric tasks, their results indirectly support our observation that ACLR‐LET patients exhibit reduced reactive strength at RTS. These patients may benefit from progressive plyometric training, including depth jumps and drop landings, to restore elastic energy transfer efficiency before RTS.

### Indications for ACLR‐LET versus ACLR alone

The findings from Weaver et al. further emphasise that athletes undergoing ACLR‐LET may require a more extended rehabilitation timeline to fully restore quadriceps strength before RTS. Given the established benefits of LET in reducing graft rupture risk [9], rehabilitation specialists must carefully balance the benefits of added rotational stability with the potential delays in neuromuscular recovery. As Weaver et al. highlight, current ACL rehabilitation protocols may not sufficiently account for these additional demands, underscoring the need for targeted neuromuscular interventions post‐ACLR‐LET.

### Study limitations

This study has several limitations. The relatively small, retrospective cohort limits generalisability, particularly to older or less active populations. Additionally, the absence of preoperative strength or RSI data prevents us from quantifying the extent of post‐surgical deficits. While significant differences in reactive strength were observed, no formal sample size calculation was conducted, and the study may have been underpowered to detect more subtle effects. Future prospective research should include a priori power analyses and baseline assessments to strengthen causal inference and external validity.

Another important limitation is the potential for selection bias due to the non‐randomised nature of group allocation. Patients who underwent ACLR‐LET were selected based on predefined high‐risk characteristics (e.g., younger age, generalised laxity, high‐grade pivot shift), which may independently influence neuromuscular performance and recovery. As such, the lower reactive strength observed in the ACLR‐LET group could reflect baseline differences rather than the surgical procedure alone. Future prospective or matched studies are needed to isolate the specific impact of LET on neuromuscular recovery.

A further limitation was the inability to control rehabilitation variability. Although all athletes followed a standardised rehabilitation protocol, physiotherapy attendance and adherence were not monitored. Differences in rehabilitation exposure may have influenced outcomes, particularly RSI scores. Future research should incorporate objective rehabilitation monitoring (e.g., wearable load‐tracking technology) to standardise training loads and track neuromuscular progression post‐ACLR.

## CONCLUSION

No significant differences in quadriceps isokinetic strength or limb symmetry index were observed between ACLR and ACLR‐LET groups at 6–8 months postoperatively. While RSI values were lower in the ACLR‐LET cohort, these differences did not reach statistical significance. Nevertheless, the observed trend suggests further investigation is warranted to explore potential neuromuscular adaptations associated with LET procedures and to clarify whether these trends have clinical relevance for RTS decisions.

## AUTHOR CONTRIBUTIONS

**Mihail Lazar Mioc**: Analysis; writing; review; editing. **Sarah Crosbie**: Conceptualisation; acquisition; investigation; writing. **Blaithin Brady**: Acquisition; investigation; supervision. **Mihai Vioreanu**: Conceptualisation; methodology supervision; review.

## CONFLICT OF INTEREST STATEMENT

The authors declare no conflicts of interest.

## ETHICS STATEMENT

This study was performed in line with the Declaration of Helsinki. Explicit consent to participate was not required as retrospective chart reviews are considered low‐risk studies under institutional guidelines, and approval from our institution's ethics committee was obtained per reference number MiVi_SSC_2021_ACLR. No funding was received during the preparation of this manuscript.

## Data Availability

Data is available on request from the authors.
